# Genome sequences of horticultural plants: past, present, and future

**DOI:** 10.1038/s41438-019-0195-6

**Published:** 2019-10-08

**Authors:** Fei Chen, Yunfeng Song, Xiaojiang Li, Junhao Chen, Lan Mo, Xingtan Zhang, Zhenguo Lin, Liangsheng Zhang

**Affiliations:** 1 College of Horticulture, Nanjing Agricultural University, Nanjing, 210095 China; 2 College of Crop Science, Fujian Agriculture and Forestry University, Fuzhou, 350002 China; 3 State Key Laboratory of Subtropical Silviculture, School of Forestry and Biotechnology, Zhejiang A&F University, Hangzhou, 311300 China; 4 Department of Biology, Saint Louis University, St. Louis, MO 63103 USA; 5 Fujian Provincial Key Laboratory of Haixia Applied Plant Systems Biology and Quality Science and Processing Technology in Special Starch, Key Laboratory of Ministry of Education for Genetics & Breeding and Multiple Utilization of Crops, College of Crop Science, Fuzhou, China

**Keywords:** Plant molecular biology, Structural variation

## Abstract

Horticultural plants play various and critical roles for humans by providing fruits, vegetables, materials for beverages, and herbal medicines and by acting as ornamentals. They have also shaped human art, culture, and environments and thereby have influenced the lifestyles of humans. With the advent of sequencing technologies, there has been a dramatic increase in the number of sequenced genomes of horticultural plant species in the past decade. The genomes of horticultural plants are highly diverse and complex, often with a high degree of heterozygosity and a high ploidy due to their long and complex history of evolution and domestication. Here we summarize the advances in the genome sequencing of horticultural plants, the reconstruction of pan-genomes, and the development of horticultural genome databases. We also discuss past, present, and future studies related to genome sequencing, data storage, data quality, data sharing, and data visualization to provide practical guidance for genomic studies of horticultural plants. Finally, we propose a horticultural plant genome project as well as the roadmap and technical details toward three goals of the project.

## Introduction

Horticultural plants mostly comprise vegetable-producing, fruit-bearing, ornamental, and beverage-producing plants and herbal medicinal plants. These plants have played important economic and social roles in the human lives and health by providing basic food needs, beautifying urban and rural landscapes, and improving personal esthetics. For example, the Food and Agriculture Organization of the United Nations reported that, while worldwide cereal food together is valued at 125 points (normalized value), vegetables and fruits together are valued at 137 points (http://faostat.fao.org). Horticultural plants also contribute to ecological balance by improving our biological environment by providing oxygen and balancing urban temperatures.

Horticultural plants are distributed among a wide variety of taxonomic plant spectra, which include a large number of flowering plants and a few early-divergent land plants. The sizes of their genomes vary greatly. For example, the vegetable garlic (*Allium sativum*) has a diploid genome (2*n* = 16) with an estimated genome size of >30 Gb^1^, and onion (*Allium cepa*) has a similar genome size^2^. In addition, most horticultural plants are domesticated, and their genome sequences have experienced strong artificial selection. For example, grape was found to have been cultivated (via viticulture) for >6000 years^3^; citrus, >4000 years^4^. In addition, some horticultural plants are intermediates of domesticated and wild plants, such as medicinal plants including ginseng (*Panax ginseng*), noto ginseng (*Panax notoginseng*), and Artemisia (*Artemisia annua*). Many domesticated horticultural plants have high levels of genetic diversity and heterozygosity, such as sunflower (10% of bases differ between homologous chromosomes)^5^, grape (7%)^6^, and potato (4.8%)^7^.

## De novo sequencing of horticultural plant genomes

**Fig. 1:**
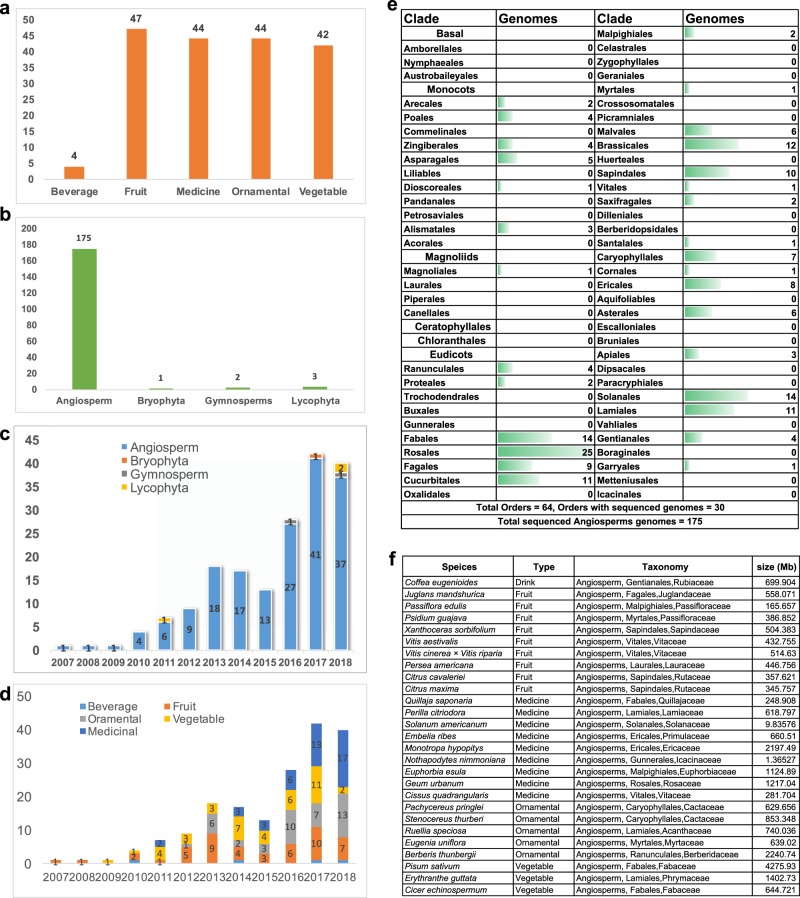
Statistics of genome-sequenced horticultural plant species. **a** Distribution of genome-sequenced horticultural plants. **b** Botanical distribution of genome-sequenced horticultural plants. **c** Annual increase in the genome-sequenced horticultural plants by botanical taxonomy. **d** Annual increase in the genome-sequenced horticultural plants by horticultural category. **e** The reported 181 horticultural plant species fall into 30 angiosperm orders. **f** List of the released but not reported horticultural plant species

As of December 31, 2018, the genomes of 181 horticultural species have been sequenced (Table 1). These include 4 beverage, 47 fruit, 44 medicinal, 44 ornamental, and 42 vegetable plants (Fig. 1a). In terms of taxonomic distribution, these plants include 175 angiosperms, 2 gymnosperms, 3 lycophytes, and 1 moss (Fig. 1b). As shown in Fig. 1c, the number of sequenced genomes of horticultural plants completed each year has significantly increased from 1 in 2007 to 40 in 2018. Although most of the horticultural plants are angiosperms, the genome sequencing of non-angiosperm species has also demonstrated steady growth (Fig. 1c). Vegetables and fruits have been a focus of plant research in the past few years. However, only two vegetables and seven fruits had their genomes sequenced in 2018 (Fig. 1d). This is probably because many economically important vegetables and fruits were already sequenced prior to 2018.

**Table 1:** List of genome-sequenced horticultural plant species and their close relatives

Species	Common name	Taxonomy	Type	DB-url
*Zoysia japonica*	Japanese lawn grass	Angiosperm/Alismatales/Poaceae	Ornamental	zoysia.kazusa.or.jp
*Zoysia matrella*	Manila grass	Angiosperm/Alismatales/Poaceae	Ornamental	zoysia.kazusa.or.jp
*Zoysia pacifica*	Mascarene grass	Angiosperm/Alismatales/Poaceae	Ornamental	zoysia.kazusa.or.jp
*Cocos nucifera*	Coconut palm	Angiosperm/Arecales/Arecaceae	Fruit	gigadb.org
*Phoenix dactylifera*	Date palm	Angiosperm/Arecales/Arecaceae	Fruit	drdb.big.ac.cn
*Asparagus officinalis*	Garden asparagus	Angiosperm/Asparagales/Asparagaceae	Vegetable	phytozome.jgi.doe.gov
*Dendrobium catenatum*	N.A.	Angiosperm/Asparagales/Orchidaceae	Medicinal	herbalplant.ynau.edu.cn
*Gastrodia elata*	Tianma	Angiosperm/Asparagales/Orchidaceae	Medicinal	herbalplant.ynau.edu.cn
*Phalaenopsis aphrodite*	Aphrodite's phalaenopsis	Angiosperm/Asparagales/Orchidaceae	Ornamental	genomevolution.org; chibba.agtec.uga.edu/duplication; orchidstra2.abrc.sinica.edu.tw
*Phalaenopsis equestris*	Horse phalaenopsis	Angiosperm/Asparagales/Orchidaceae	Ornamental	genomevolution.org; chibba.agtec.uga.edu/duplication; orchidstra2.abrc.sinica.edu.tw
*Dioscorea rotundata*	White Guinea yam	Angiosperm/Dioscoreales/Dioscoreaceae	Vegetable	genomevolution.org/CoGe; plants.ensembl.org
*Ananas comosus*	Pineapple	Angiosperm/Poales/Bromeliaceae	Fruit	phytozome.jgi.doe.gov; genomevolution.org/CoGe; pineapple.angiosperms.org/pineapple/html/index.html
*Echinochloa crus-galli*	Cockspur grass	Angiosperm/Poales/Poaceae	Medicinal	horticulture.eplant.org
*Lolium perenne*	Perennial ryegrass	Angiosperm/Poales/Poaceae	Ornamental	pgsb.helmholtz-muenchen.de
*Zizania latifolia*	Jiaobai	Angiosperm/Poales/Poaceae	Vegetable	plants.ensembl.org
*Musa acuminata*	Wild banana	Angiosperm/Zingiberales/Musaceae	Ornamental	chibba.agtec.uga.edu/duplication; plants.ensembl.org/; phytozome.jgi.doe.gov; banana-genome-hub.southgreen.fr
*Musa balbisiana*	Wild banana	Angiosperm/Zingiberales/Musaceae	Ornamental	banana-genome-hub.southgreen.fr
*Musa itinerans*	Yunnan banana	Angiosperm/Zingiberales/Musaceae	Fruit	banana-genome-hub.southgreen.fr
*Ensete ventricosum*	Ethiopian banana	Angiosperm/Zingiberales/Musaceae	Medicinal	horticulture.eplant.org
*Liriodendron chinense*	Chinese tulip tree	Angiosperm/Magnoliales/Magnoliaceae	Ornamental	www.hardwoodgenomics.org
*Manihot esculenta*	Cassava	Angiosperm/Malpighiales/Euphorbiaceae	Vegetable	genomevolution.org/CoGe; bioinformatics.psb.ugent.be/plaza; plants.ensembl.org; www.plantgdb.org/; phytozome.jgi.doe.gov
*Rhizophora apiculata*	Tall-stilt mangrove	Angiosperm/Malpighiales/Rhizophoraceae	Medicinal	genomevolution.org/coge
*Begonia fuchsioides*	Shrub Begonia	Angiosperm/Cucurbitales/Begoniaceae	Ornamental	
*Cucumis melo*	Muskmelon	Angiosperm/Cucurbitales/Cucurbitaceae	Fruit	cucurbitgenomics.org/; bioinformatics.psb.ugent.be/plaza
*Citrullus lanatus*	Watermelon	Angiosperm/Cucurbitales/Cucurbitaceae	Fruit	www.coolseasonFoodlegume.org; bioinformatics.psb.ugent.be/plaza; chibba.agtec.uga.edu/duplication; cucurbitgenomics.org
*Siraitia grosvenorii*	Monk fruit	Angiosperm/Cucurbitales/Cucurbitaceae	Medicinal	herbalplant.ynau.edu.cn
*Cucumis sativus*	Cucumber	Angiosperm/Cucurbitales/Cucurbitaceae	Vegetable	genomevolution.org/CoGe; bioinformatics.psb.ugent.be/plaza; phytozome.jgi.doe.gov; chibba.agtec.uga.edu/duplication; plants.ensembl.org; www.plantgdb.org; cucurbitgenomics.org
*Cucurbita argyrosperma*	Silver-seed gourd	Angiosperm/Cucurbitales/Cucurbitaceae	Vegetable	cucurbitgenomics.org
*Cucurbita maxima*	Winter squash	Angiosperm/Cucurbitales/Cucurbitaceae	Vegetable	cucurbitgenomics.org
*Cucurbita moschata*	Pumpkin	Angiosperm/Cucurbitales/Cucurbitaceae	Vegetable	cucurbitgenomics.org
*Cucurbita pepo*	Summer squash	Angiosperm/Cucurbitales/Cucurbitaceae	Vegetable	cucurbitgenomics.org
*Lagenaria siceraria*	Bottle gourd	Angiosperm/Cucurbitales/Cucurbitaceae	Vegetable	genomevolution.org; cucurbitgenomics.org
*Momordica charantia*	Bitter melon	Angiosperm/Cucurbitales/Cucurbitaceae	Vegetable	
*Glycyrrhiza uralensis*	Chinese liquorice	Angiosperm/Fabales/Fabaceae	Medicinal	ngs-data-archive.psc.riken.jp
*Trifolium pratense*	Red clover	Angiosperm/Fabales/Fabaceae	Medicinal	http://www.cacaogenomedb.org; bioinformatics.psb.ugent.be/plaza; plants.ensembl.org; phytozome.jgi.doe.gov
*Cercis canadensis*	Eastern redbud	Angiosperm/Fabales/Fabaceae	Ornamental	genomevolution.orgauth.iplantc.org
*Lupinus angustifolius*	Narrow-leaved lupine	Angiosperm/Fabales/Fabaceae	Ornamental	plants.ensembl.org
*Mimosa pudica*	Sensitive plant	Angiosperm/Fabales/Fabaceae	Ornamental	www.medicagogenome.org
*Cajanus cajan*	Pigeon pea	Angiosperm/Fabales/Fabaceae	Vegetable	brassicadb.org/brad; genomevolution.org/CoGe; bioinformatics.psb.ugent.be/plaza; chibba.agtec.uga.edu/duplication
*Cicer arietinum*	Chick pea	Angiosperm/Fabales/Fabaceae	Vegetable	genomevolution.org/CoGe; bioinformatics.psb.ugent.be/plaza; chibba.agtec.uga.edu/duplication; phytozome.jgi.doe.gov
*Cicer reticulatum*	Chick pea	Angiosperm/Fabales/Fabaceae	Vegetable	www.coolseasonfoodlegume.org
*Glycine max*	Soybean	Angiosperm/Fabales/Fabaceae	Vegetable	genomevolution.org/CoGe; bioinformatics.psb.ugent.be/plaza; phytozome.jgi.doe.gov; chibba.agtec.uga.edu/duplication/; plants.ensembl.org; www.plantgdb.org
*Medicago truncatula*	Barrelclover	Angiosperm/Fabales/Fabaceae	Vegetable	phytozome.jgi.doe.gov; bioinformatics.psb.ugent.be/plaza; chibba.agtec.uga.edu/duplication; /plant/plantsdb.jsp; plants.ensembl.org; www.plantgdb.org
*Phaseolus vulgaris*	Common bean	Angiosperm/Fabales/Fabaceae	Vegetable	genomevolution.org/CoGe; chibba.agtec.uga.edu/duplication; plants.ensembl.org; phytozome.jgi.doe.gov
*Vicia faba*	Fava bean	Angiosperm/Fabales/Fabaceae	Vegetable	
*Vigna angularis*	Adzuki bean	Angiosperm/Fabales/Fabaceae	Vegetable	plants.ensembl.org
*Vigna radiata*	Mungbean	Angiosperm/Fabales/Fabaceae	Vegetable	plants.ensembl.org
*Casuarina equisetifolia*	Australian pine tree	Angiosperm/Fagales/Casuarinaceae	Ornamental	hardwoodgenomics.org
*Castanea mollissima*	Chinese chestnut	Angiosperm/Fagales/Fagaceae	Fruit	genomevolution.org/CoGe
*Juglans cathayensis*	Chinese walnut	Angiosperm/Fagales/Juglandaceae	Fruit	www.hardwoodgenomics.org
*Juglans hindsii*	Northern California walnut	Angiosperm/Fagales/Juglandaceae	Fruit rootstock	www.hardwoodgenomics.org
*Juglans microcarpa*	Texas black walnut	Angiosperm/Fagales/Juglandaceae	Fruit	www.hardwoodgenomics.org
*Juglans nigra*	Eastern black walnut	Angiosperm/Fagales/Juglandaceae	Fruit rootstock	www.hardwoodgenomics.org
*Juglans regia*	Common walnut	Angiosperm/Fagales/Juglandaceae	Fruit	www.hardwoodgenomics.org
*Juglans sigillata*	Iron walnut	Angiosperm/Fagales/Juglandaceae	Fruit	www.hardwoodgenomics.org
*Morella rubra*	Red bayberry	Angiosperm/Fagales/Myricaceae	Fruit	
*Nelumbo nucifera*	Sacred lotus	Angiosperm/Proteales/Nelumbonaceae	Ornamental	bioinformatics.psb.ugent.be/plaza; chibba.agtec.uga.edu/duplication
*Macadamia integrifolia*	Macadamia nut	Angiosperm/Proteales/Proteaceae	Fruit	www.hardwoodgenomics.org
*Macleaya cordata*	Plume poppy	Angiosperm/Ranunculales/Papaveraceae	Medicinal	herbalplant.ynau.edu.cn
*Papaver somniferum*	Opium poppy	Angiosperm/Ranunculales/Papaveraceae	Medicinal	genomevolution.orgauth.iplantc.org
*Eschscholzia californica*	California poppy	Angiosperm/Ranunculales/Papaveraceae	Ornamental	eschscholzia.kazusa.or.jp
*Aquilegia coerulea*	Colorado blue columbine	Angiosperm/Ranunculales/Ranunculaceae	Medicinal	genome.jgi.doe.gov; genomevolution.org/CoGe; phytozome.jgi.doe.gov
*Cannabis sativa*	Hemp	Angiosperm/Rosales/Cannabaceae	Medicinal	genome.ccbr.utoronto.ca
*Parasponia andersonii*	Caoye shanhuangma	Angiosperm/Rosales/Cannabaceae	Medicinal	www.bioinformatics.nl/parasponia
*Trema orientalis*	Indian charcoal tree	Angiosperm/Rosales/Cannabaceae	Medicinal	www.bioinformatics.nl/parasponia
*Artocarpus camansi*	Breadnut	Angiosperm/Rosales/Moraceae	Fruit	sites.northwestern.edu/zerega-lab/research/artocarpus-genomics
*Ficus carica*	Common fig	Angiosperm/Rosales/Moraceae	Fruit	
*Ziziphus jujuba*	Jujube	Angiosperm/Rosales/Rhamnaceae	Fruit	genomevolution.org/CoGe; bioinformatics.psb.ugent.be/plaza
*Fragaria iinumae*	Nogo strawberry	Angiosperm/Rosales/Rosaceae	Fruit	strawberry-garden.kazusa.or.jp
*Fragaria nipponica*	Japanese strawberry	Angiosperm/Rosales/Rosaceae	Fruit	strawberry-garden.kazusa.or.jp
*Fragaria nubicola*	Tibet strawberry	Angiosperm/Rosales/Rosaceae	Fruit	strawberry-garden.kazusa.or.jp
*Fragaria orientalis*	Eastern strawberry	Angiosperm/Rosales/Rosaceae	Fruit	strawberry-garden.kazusa.or.jp
*Fragaria vesca*	Woodland strawberry	Angiosperm/Rosales/Rosaceae	Fruit	strawberry-garden.kazusa.or.jp; genomevolution.org/CoGe;bioinformatics.psb.ugent.be/plaza; chibba.agtec.uga.edu/duplication; phytozome.jgi.doe.gov
*Fragaria* × *ananassa*	Strawberry	Angiosperm/Rosales/Rosaceae	Fruit	strawberry-garden.kazusa.or.jp
*Malus domestica*	Apple	Angiosperm/Rosales/Rosaceae	Fruit	genomevolution.org/CoGe; bioinformatics.psb.ugent.be/plaza; phytozome.jgi.doe.gov; www.rosaceae.org
*Morus notabilis*	Mulberry	Angiosperm/Rosales/Rosaceae	Fruit	morus.swu.edu.cn
*Prunus avium*	Sweet cherry	Angiosperm/Rosales/Rosaceae	Fruit	www.rosaceae.org
*Prunus persica*	Peach	Angiosperm/Rosales/Rosaceae	Fruit	genomevolution.org/CoGe; bioinformatics.psb.ugent.be/plaza; chibba.agtec.uga.edu/duplication; plants.ensembl.org; phytozome.jgi.doe.gov
*Pyrus bretschneideri*	Chinese pear	Angiosperm/Rosales/Rosaceae	Fruit	bioinformatics.psb.ugent.be/plaza; chibba.agtec.uga.edu/duplication
*Pyrus communis*	European pear	Angiosperm/Rosales/Rosaceae	Fruit	www.rosaceae.org
*Rubus occidentalis*	Black raspberry	Angiosperm/Rosales/Rosaceae	Fruit	www.rosaceae.org
*Prunus mume*	Mei	Angiosperm/Rosales/Rosaceae	Ornamental	genomevolution.org/CoGe; chibba.agtec.uga.edu/duplication
*Prunus yedoensis*	Yoshino cherry	Angiosperm/Rosales/Rosaceae	Ornamental	www.rosaceae.org
*Rosa* × *damascena*	Damask rose	Angiosperm/Rosales/Rosaceae	Ornamental	gigadb.org; www.rosaceae.org
*Rosa chinensis*	Chinese rose	Angiosperm/Rosales/Rosaceae	Ornamental	www.rosaceae.org
*Rosa multiflora*	Many-flowered rose	Angiosperm/Rosales/Rosaceae	Ornamental	www.rosaceae.org
*Rosa roxburghii*	Chestnut rose	Angiosperm/Rosales/Rosaceae	Ornamental	www.rosaceae.org
*Daucus carota*	Carrot	Angiosperm/Apiales/Apiaceae	Vegetable	bioinformatics.psb.ugent.be/plaza; plants.ensembl.org; phytozome.jgi.doe.gov
*Panax ginseng*	Asian ginseng	Angiosperm/Apiales/Araliaceae	Medicinal	herbalplant.ynau.edu.cn
*Panax notoginseng*	Sanchi ginseng	Angiosperm/Apiales/Araliaceae	Medicinal	herbalplant.ynau.edu.cn
*Artemisia annua*	Sweet wormwood	Angiosperm/Asterales/Asteraceae	Medicinal	herbalplant.ynau.edu.cn
*Conyza canadensis*	Horseweed	Angiosperm/Asterales/Asteraceae	Medicinal	genomevolution.org/CoGe
*Erigeron breviscapus*	Chinese fleabane	Angiosperm/Asterales/Asteraceae	Medicinal	www.ncbi.nlm.nih.gov/genome/?term=Eleusine+coracana
*Chrysanthemum nankingense*	Juhuanao	Angiosperm/Asterales/Asteraceae	Vegetable	genomevolution.org/CoGe
*Cynara cardunculus*	Cardoon	Angiosperm/Asterales/Asteraceae	Vegetable	www.artichokegenome.unito.it
*Lactuca sativa*	Lettuce	Angiosperm/Asterales/Asteraceae	Vegetable	phytozome.jgi.doe.gov
*Eutrema yunnanense*	Shan yu cai	Angiosperm/Brassicales/Brassicaceae	Medicinal	
*Lepidium meyenii*	Maca	Angiosperm/Brassicales/Brassicaceae	Medicinal	maca.eplant.org
*Brassica juncea*	Zhacai	Angiosperm/Brassicales/Brassicaceae	Vegetable	brassicadb.org
*Brassica oleracea*	Cabbage	Angiosperm/Brassicales/Brassicaceae	Vegetable	brassicadb.org; genomevolution.org/CoGe; bioinformatics.psb.ugent.be/plaza; chibba.agtec.uga.edu/duplication; plants.ensembl.org
*Brassica rapa*	Chinese cabbage	Angiosperm/Brassicales/Brassicaceae	Vegetable	plants.ensembl.org; genomevolution.org/CoGe; bioinformatics.psb.ugent.be/plaza; phytozome.jgi.doe.gov; chibba.agtec.uga.edu/duplication; plants.ensembl.org; www.plantgdb.org
*Capsella bursa-pastoris*	Shepherd’s purse	Angiosperm/Brassicales/Brassicaceae	Vegetable	genome.ccbr.utoronto.ca/cgi-bin/hgGateway
*Capsella rubella*	Red shepherd’s purse	Angiosperm/Brassicales/Brassicaceae	Vegetable	genomevolution.org/CoGe; bioinformatics.psb.ugent.be/plaza; chibba.agtec.uga.edu/duplication; phytozome.jgi.doe.gov
*Raphanus sativus*	Radish	Angiosperm/Brassicales/Brassicaceae	Vegetable	radish.kazusa.or.jp
*Thlaspi arvense*	Field pennycress	Angiosperm/Brassicales/Brassicaceae	Vegetable	pennycress.umn.edu
*Carica papaya*	Papaya	Angiosperm/Brassicales/Caricaceae	Fruit	genomevolution.org/CoGe; bioinformatics.psb.ugent.be/plaza; phytozome.jgi.doe.gov; chibba.agtec.uga.edu/duplication; www.plantgdb.org
*Tarenaya hassleriana*	Spider flower	Angiosperm/Brassicales/Cleomaceae	Ornamental	genomevolution.org/CoGe; bioinformatics.psb.ugent.be/plaza
*Moringa oleifera*	Moringa	Angiosperm/Brassicales/Moringaceae	Vegetable	bioinformatics.psb.ugent.be/plaza
*Amaranthus hypochondriacus*	Prince’s feather	Angiosperm/Caryophyllales/Amaranthaceae	Ornamental	phytozome.jgi.doe.gov; genomevolution.org/CoGe; bioinformatics.psb.ugent.be/plaza
*Beta vulgaris*	Sugar beet	Angiosperm/Caryophyllales/Amaranthaceae	Vegetable	bioinformatics.psb.ugent.be/plaza; chibba.agtec.uga.edu/duplication; plants.ensembl.org
*Spinacia oleracea*	Spinach	Angiosperm/Caryophyllales/Amaranthaceae	Vegetable	spinachbase.org
*Carnegiea gigantea*	Saguaro cactus	Angiosperm/Caryophyllales/Cactaceae	Ornamental	phytozome.jgi.doe.gov
*Dianthus caryophyllus*	Carnation	Angiosperm/Caryophyllales/Caryophyllaceae	Ornamental	carnation.kazusa.or.jp
*Casuarina glauca*	Swamp oak	Angiosperm/Caryophyllales/Casuarinaceae	Ornamental	
*Drosera capensis*	Cape sundew	Angiosperm/Caryophyllales/Droseraceae	Ornamental	
*Camptotheca acuminata*	Happy tree	Angiosperm/Cornales/Nyssaceae	Ornamental	www.plantkingdomgdb.com; genomevolution.org/CoGe
*Actinidia chinensis*	Kiwifruit	Angiosperm/Ericales/Actinidiaceae	Fruit	bdg.hfut.edu.cn/kir; genomevolution.org/coge
*Diospyros lotus*	Date-plum	Angiosperm/Ericales/Ebenaceae	Fruit	gigadb.org
*Vaccinium corymbosum*	Blueberry	Angiosperm/Ericales/Ericaceae	Fruit	www.vaccinium.org
*Vaccinium macrocarpon*	American cranberry	Angiosperm/Ericales/Ericaceae	Fruit	gigadb.org
*Rhododendron delavayi*	Tree rhododendron	Angiosperm/Ericales/Ericaceae	Ornamental	
*Primula vulgaris*	Common primrose	Angiosperm/Ericales/Primulaceae	Medicinal	phytozome.jgi.doe.gov
*Primula veris*	Cowslip	Angiosperm/Ericales/Primulaceae	Ornamental	plantgenie.org
*Camellia sinensis*	Tea tree	Angiosperm/Ericales/Theaceae	Beverage	tpia.teaplant.org
*Eucommia ulmoides*	Hardy rubber tree	Angiosperm/Garryales/Eucommiaceae	Medicinal	
*Calotropis gigantea*	Crown flower	Angiosperm/Gentianales/Apocynaceae	Medicinal	
*Catharanthus roseus*	Madagascar periwinkle	Angiosperm/Gentianales/Apocynaceae	Medicinal	genomevolution.org/CoGe
*Coffea arabica*	Arabian coffee	Angiosperm/Gentianales/Rubiaceae	Beverage	www.coffee-genome.org; phytozome.jgi.doe.gov
*Coffea canephora*	Robusta Coffee	Angiosperm/Gentianales/Rubiaceae	Beverage	genomevolution.org/CoGe; www.coffee-genome.org; bioinformatics.psb.ugent.be/plaza
*Andrographis paniculata*	Green chireta	Angiosperm/Lamiales/Acanthaceae	Medicinal	
*Handroanthus impetiginosus*	Pink trumpet tree	Angiosperm/Lamiales/Bignoniaceae	Ornamental	www.hardwoodgenomics.org
*Boea hygrometrica*	N.A.	Angiosperm/Lamiales/Gesneriaceae	Ornamental	genomevolution.org
*Mentha longifolia*	Horse mint	Angiosperm/Lamiales/Lamiaceae	Medicinal	phytozome.jgi.doe.gov
*Ocimum sanctum*	Holy basil	Angiosperm/Lamiales/Lamiaceae	Medicinal	caps.ncbs.res.in/Ote
*Scutellaria baicalensis*	Baikal skullcap	Angiosperm/Lamiales/Lamiaceae	Medicinal	
*Lavandula angustifolia*	Lavender	Angiosperm/Lamiales/Lamiaceae	Ornamental	
*Salvia splendens*	Scarlet sage	Angiosperm/Lamiales/Lamiaceae	Ornamental	gigadb.org
*Osmanthus fragrans*	Sweet osmanthus	Angiosperm/Lamiales/Oleaceae	Medicinal	sweetolive.eplant.org
*Fraxinus excelsior*	European ash	Angiosperm/Lamiales/Oleaceae	Ornamental	www.hardwoodgenomics.org
*Mimulus guttatus*	Seep monkeyflower	Angiosperm/Lamiales/Phrymaceae	Ornamental	phytozome.jgi.doe.gov; www.plantgdb.org
*Theobroma cacao*	Cacao	Angiosperm/Malvales/Malvaceae	Beverage	bioinformatics.psb.ugent.be/plaza/; chibba.agtec.uga.edu/duplication; plants.ensembl.org; phytozome.jgi.doe.gov
*Durio zibethinus*	Durian	Angiosperm/Malvales/Malvaceae	Fruit	
*Corchorus olitorius*	Chang shuo huang ma	Angiosperm/Malvales/Malvaceae	Medicinal	bioinformatics.psb.ugent.be/plaza
*Bombax ceiba*	Red silk-cotton tree	Angiosperm/Malvales/Malvaceae	Ornamental	
*Hibiscus syriacus*	Rose of Sharon	Angiosperm/Malvales/Malvaceae	Ornamental	
*Aquilaria agallocha*	Agarwood	Angiosperm/Malvales/Thymelaeaceae	Medicinal	
*Santalum album*	Indian sandalwood	Angiosperm/Santalales/Santalaceae	Medicinal	
*Citrus clementina*	Clementine citrus	Angiosperm/Sapindales/Rutaceae	Fruit	genomevolution.org/CoGe; bioinformatics.psb.ugent.be/plaza; phytozome.jgi.doe.gov
*Citrus grandis*	Pummelo	Angiosperm/Sapindales/Rutaceae	Fruit	www.citrusgenomedb.org
*Citrus ichangensis*	Ichang papeda	Angiosperm/Sapindales/Rutaceae	Fruit	www.citrusgenomedb.org
*Citrus paradisi* × *Poncirus trifoliata*	Citrumelo	Angiosperm/Sapindales/Rutaceae	Fruit	
*Citrus reticulata*	Mandarin orange	Angiosperm/Sapindales/Rutaceae	Fruit	www.citrusgenomedb.org
*Citrus sinensis*	Sweet orange	Angiosperm/Sapindales/Rutaceae	Fruit	www.citrusgenomedb.org
*Citrus unshiu*	Cold hardy mandarin	Angiosperm/Sapindales/Rutaceae	Fruit	www.citrusgenomedb.org
*Atalantia buxifolia*	Jiu bing le	Angiosperm/Sapindales/Rutaceae	Medicinal	www.citrusgenomedb.org
*Citrus medica*	Citron	Angiosperm/Sapindales/Rutaceae	Medicinal	www.citrusgenomedb.org
*Dimocarpus longan*	Longan	Angiosperm/Sapindales/Sapindaceae	Fruit	gigadb.org
*Rhodiola crenulata*	Tibetan Rhodiola	Angiosperm/Saxifragales/Crassulaceae	Medicinal	gigadb.org
*Kalanchoe fedtschenkoi*	Lavender-scallops	Angiosperm/Saxifragales/Crassulaceae	Ornamental	phytozome.jgi.doe.gov
*Cuscuta australis*	Australian dodder	Angiosperm/Solanales/Convolvulaceae	Medicinal	
*Cuscuta campestris*	Prairie dodder	Angiosperm/Solanales/Convolvulaceae	Medicinal	plabipd.de/project_cuscuta2/start.ep
*Ipomoea nil*	Japanese morning glory	Angiosperm/Solanales/Convolvulaceae	Ornamental	viewer.shigen.info/asagao
*Nicotiana sylvestris*	Flowering tobacco	Angiosperm/Solanales/Solanaceae	Ornamental	solgenomics.net
*Petunia axillaris*	N.A.	Angiosperm/Solanales/Solanaceae	Ornamental	genome.jgi.doe.gov; bioinformatics.psb.ugent.be/plaza; solgenomics.net
*Petunia inflata*	N.A.	Angiosperm/Solanales/Solanaceae	Ornamental	solgenomics.net
*Solanum pennellii*	Wild tomato	Angiosperm/Solanales/Solanaceae	Vegetable relative	
*Capsicum annuum*	Spanish pepper	Angiosperm/Solanales/Solanaceae	Vegetable	bioinformatics.psb.ugent.be/plaza; chibba.agtec.uga.edu/duplication; solgenomics.net
*Capsicum baccatum*	Berry-like pepper	Angiosperm/Solanales/Solanaceae	Vegetable	genomevolution.org/CoGe
*Capsicum chinense*	Bonnet pepper	Angiosperm/Solanales/Solanaceae	Vegetable	www.pepperpan.org:8012
*Solanum lycopersicum*	Tomato	Angiosperm/Solanales/Solanaceae	Vegetable	bioinformatics.psb.ugent.be/plaza; phytozome.jgi.doe.gov; chibba.agtec.uga.edu/duplication;pgsb.helmholtz-muenchen.de; plants.ensembl.org; www.plantgdb.org; solgenomics.net
*Solanum melongena*	Eggplant	Angiosperm/Solanales/Solanaceae	Vegetable	solgenomics.net; genomevolution.org/CoGe
*Solanum pimpinellifolium*	Currant tomato	Angiosperm/Solanales/Solanaceae	Vegetable	solgenomics.net
*Solanum tuberosum*	Potato	Angiosperm/Solanales/Solanaceae	Vegetable	genomevolution.org/CoGe; bioinformatics.psb.ugent.be/plaza; chibba.agtec.uga.edu/duplication; plants.ensembl.org; www.plantgdb.org; phytozome.jgi.doe.gov; solgenomics.net
*Vitis vinifera*	Grape	Angiosperm/Vitales/Vitaceae	Fruit	phytozome.jgi.doe.gov; genomevolution.org/CoGe; bioinformatics.psb.ugent.be/plaza; chibba.agtec.uga.edu/duplication; plants.ensembl.org; www.plantgdb.org
*Punica granatum*	Pomegranate	Angiosperm/Myrtales/Lythraceae	Fruit	www.hardwoodgenomics.org
*Marchantia polymorpha*	Umbrella liverwort	Bryophyta/Marchantiales/Marchantiaceae	Medicinal	bioinformatics.psb.ugent.be/plaza; phytozome.jgi.doe.gov
*Ginkgo biloba*	Ginkgo tree	Gymnosperms/Ginkgoales/Ginkgoaceae	Medicinal	gigadb.org/site/index
*Gnetum montanum*	Jointfir	Gymnosperms/Gnetales/Gnetaceae	Medicinal	www.datadryad.org/resource/doi:10.5061/dryad.0vm37; genomevolution.org/coge
*Selaginella lepidophylla*	Resuscitation moss	Lycophyta/Selaginellales/Selaginellaceae	Medicinal	plantgdb.org/SmGDB/
*Selaginella moellendorffii*	Spikemoss	Lycophyta/Selaginellales/Selaginellaceae	Medicinal	phytozome.jgi.doe.gov; genomevolution.org/CoGe; chibba.agtec.uga.edu/duplication; bioinformatics.psb.ugent.be/plaza; plants.ensembl.org; www.plantgdb.org/
*Selaginella tamariscina*	Little club moss	Lycophyta/Selaginellales/Selaginellaceae	Medicinal	www.ncbi.nlm.nih.gov/assembly/GCA_003024785.1; genomevolution.org/coge

*N.A.* not available

Some angiosperms have a significant role in the economy^8^. The 181 horticultural plants with sequenced genomes are distributed in 30 of the 64 angiosperm orders. Among these 30 orders, 7 (Fabales, Rosales, Cucurbitales, Brassicales, Sapindales, Solanales, and Laminales) have >10 species whose genomes have been sequenced (Fig. 1e), suggesting their vital importance to humans.

Most of the genome-sequenced plants fall into the Rosaceae family, which is a medium-sized family with approximately 4800 species (http://www.theplantlist.org), including many popular fruit-bearing and ornamental plants. The genome-decoded fruit-producing species include breadnut (*Artocarpus camansi*)^9^, ficus (*Ficus carica*)^10^, jujube (*Ziziphus jujuba*)^11^, strawberry and its close relatives (*Fragaria* × *ananassa*, *Fragaria iinumae*, *Fragaria nipponica*, *Fragaria nubicola*, *Fragaria orientalis*, *Fragaria vesca*)^12–14^, apple (*Malus domestica*)^15^, morus (*Morus notabilis*)^16^, sweet cherry (*Prunus avium*)^17^, peach (*Prunus persica*)^18^, Chinese pear (*Pyrus bretschneideri*)^19^, European pear (*Pyrus communis*)^20^, and black raspberry (*Rubus occidentalis*)^21^. The genome-decoded ornamentals include mei (*Prunus mume*)^22^, sakura (*Prunus yedoensis*)^23^, and rose and its close relatives (*Rosa* × *damascene*, *Rosa chinensis*, *Rosa multiflora*, and *Rosa roxburghii*)^24–26^. However, the genomes of many important fruit-bearing Rosales plants, such as *Crataegus pinnatifida*, *Malus prunifolia*, *Eriobotrya japonica*, *Armeniaca vulgaris*, and *Prunus salicina*, and of Rosales ornamentals, such as *Photinia serrulata*, *Spiraea thunbergii*, *Cotoneaster multiflorus*, and *Rubus japonicas*, have not yet been sequenced. The available genome sequences of Rosales species have largely improved our understanding of the biology of fruits and flowers. For example, the high-quality apple genome sequence showed that a single allele is responsible for red fruit peal coloration^27^, and the reference genome of rose has provided insights into the floral color and scent pathways^25^.

The Solanaceae family consists of ~2700 species (http://www.theplantlist.org) that include a number of vegetable, medicinal, and ornamental species. The genomes of several important Solanaceae vegetable species have been sequenced, such as tomato (*Solanum lycopersicum*, *Solanum pimpinellifolium*)^28,29^, potato (*Solanum tuberosum*)^30^, pepper (*Capsicum annuum*, *Capsicum baccatum*, *Capsicum chinense*)^31–33^, and eggplant (*Solanum melongena*)^34^. Solanaceae ornamentals include ivy morning glory (*Ipomoea nil*)^35^, ornamental tobacco (*Nicotiana sylvestris*)^36^, and petunia (*Petunia axillaris*, *Petunia inflate*)^37^. Although these genomes have helped to understand the evolution of Solanaceae plants, additional Solanaceae horticultural genomes need to be sequenced. These include the sequences of the medicinal plants *Datura arborea*, *Datura metel*, and *Datura innoxia* and the ornamentals *Petunia* spp., *Nicotiana* spp., *Lycium* spp., *Solanum* spp., *Cestrum* spp., *Calibrachoa* spp., and *Solandra* spp. These available genome sequences have helped to decipher the evolution and genomic basis of metabolites such as vitamin C (or ascorbic acid)^38^ in tomato and alkaloids in tobacoo^39^.

The Fabaceae family, consisting of ~19,000 known species, is the third largest angiosperm family by number of species richness, followed by the Orchidaceae and Asteraceae families. Although only dozens of Fabaceae genomes have been sequenced^8^, many of them are from horticultural species. The genome-decoded Fabaceae vegetable plants include pigeon pea (*Cajanus cajan*)^40^, chickpea and its relative (*Cicer arietinum*, *Cicer reticulatum*)^41,42^, soybean (*Glycine max*)^43^, barrelclover (*Medicago truncatula*)^44^, common bean (*Phaseolus vulgaris*)^45^, faba bean (*Vicia faba*)^46^, adzuki bean (*Vigna angularis*)^47^, and mung bean (*Vigna radiata*)^48^. The genome-sequenced Fabaceae ornamentals include eastern redbud (*Cercis canadensis*)^49^, narrowleaf lupin (*Lupinus angustifolius*)^50^, and mimosa (*Mimosa pudica*). The Fabaceae medicinal plants with sequenced genomes include Chinese uralensis (*Glycyrrhiza uralensis*)^51^ and red clover (*Trifolium pratense*)^52^. Legumes are considered a valuable source of food in the future^53^; thus the sequencing of their genomes would be valuable. Determining the genomic basis of legume–rhizobium interactions would help not only to solve a classic fundamental problem in biology but also to improve nitrogen utilization in horticultural plants.

The Brassicaceae family is a medium-sized family with ~4000 species, including many horticultural plant species. The Brassicaceae vegetable plants with sequenced genomes include Zhacai (*Brassica juncea*)^54^, cabbage (*Brassica oleracea*)^55^, napa cabbage (*Brassica rapa*)^56^, Capsella (*Capsella bursa-pastoris* and *Capsella rubella*)^57,58^, radish (*Raphanus sativus*)^59^, and field pennycress (*Thlaspi arvense*)^60^. The genomes of the Brassicaceae medicinal plants *Eutrema yunnanense*^61^ and maca (*Lepidium meyenii*)^62^ have also been sequenced. With these genome sequences at hand, the genomic features of common ancestors and the subsequent evolution of the Brassicaceae can be clarified, such as the intron evolution within the Brassicaceae^63^, and gene and genome duplication events within the Brassicaceae^64,65^. These genomes would also shed light on the evolution of the hypocotyl, as has been reported in maca^62^ and radish^59^. Within the Brassicaceae family, we could foresee a growing demand for the genome sequencing of horticultural Brassicaceae plants, both for evolutionary research and for decoding the molecular basis of economically important traits.

The Cucurbitaceae family includes >3700 species belonging to 134 genera (www.theplantlist.org). Within this family, the genome-decoded vegetable plants include silver-seed gourd (*Cucurbita argyrosperma*)^66^, winter squash (*Cucurbita maxima*)^67^, pumpkin (*Cucurbita moschata*)^67^, summer squash (*Cucurbita pepo*)^68^, bottle gourd (*Lagenaria siceraria*)^69^, and bitter melon (*Momordica charantia*)^70^. The genome-decoded fruit species include muskmelon (*Cucumis melo*)^71^ and watermelon (*Citrullus lanatus*)^72^. The only genome-decoded medicinal plant is monk fruit (*Siraitia grosvenorii*)^73,74^. Via analysis of these available genome sequences, it was found that a tetraploid-inducing event occurred in the last common ancestor of the Cucurbitaceae species^75^. These genome sequences can also help to better understand the domestication history^76^ and fruit development^77^. Increasing numbers of the wild relatives of these economically important crop species, as well as those of thousands of plant cultivars, will be sequenced in the near future, providing additional details and surprises.

The Rutaceae or citrus family consists of 158 genera and 6686 species (www.theplatlist.org). The Rutaceae fruit-bearing plants with sequenced genomes include clementine (*Citrus clementina*)^78^, pomelo (*Citrus grandis*)^79^, Ichang papeda (*Citrus ichangensis*)^79^, citrumelo (*Citrus paradisi* × *Poncirus trifoliate*)^80^, mandarin orange (Citrus reticulata)^81^, sweet orange (*Citrus sinensis*)^82^, and cold-hardy mandarin (*Citrus unshiu*)^83^. The Rutaceae medicinal plants with sequenced genomes include jiu bing le (*Atalantia buxifolia*)^79^ and citron (*Citrus medica*)^79^. Via analysis of these genome sequences, the evolutionary origin and evolutionary changes in the *Citrus* genus during domestication were mapped^84^. In the future, the genome sequences of Rutaceae fruit-bearing plants including lemon (*Citrus limon*), calamansi (*Citrofortunella microcarpa*), lime (*Citrus* spp. hybrids), kumquat (*Citrus japonica*), and grapefruit (*Citrus* × *paradisi*) will require genome sequencing.

## Genome resequencing and the pan-genome of horticultural plants

A single reference genome sequence is not sufficient for identifying the best candidate genes for molecular breeding or for understanding the genomic background of a population due to the prevalence of genomic structural variations. Compared to the construction of a reference genome, genome resequencing usually requires less sequencing coverage. It is feasible to obtain a high-quality resequenced genome via mapping to a reference genome. A pan-genome is the summary of genomes of a species obtained by comparing a large number of resequenced genomes of a species or, occasionally, a genus. A pan-genome can help to understand the size of a core genome (defined as the conserved part among the related genomes), the size of a pan-genome, and the amount and nature of variations within a species or a genus, which improve our understanding of the evolution of a species/genu, as well as of agronomic traits. Currently, a growing number of pan-genomes among horticultural plants have been constructed (Table 2).

**Table 2:** Pan-genome information of horticultural plants

Pan-genome	Covered population	Year of release	Horticultural category	Tool	Pan-genome database
*Glycine soja*	7 cultivars	2014	Wild relatives of vegetable	N.A.	N.A.
*Brassica oleracea*	9 cultivars	2016	Vegetable	Gbrowse, BLAST, Gbrowse	http://brassicagenome.net/
*Capsicum* spp.	383 cultivars, including 355 *C. annuum*, four *C. baccatum*, 11 *C. chinense*, 13 *C. frutescens*	2018	Vegetable	Search, Jbrowse	http://www.pepperpan.org:8012/
*Helianthus annuus*	493 accessions	2018	Ornamental	N.A.	www.sunflowergenome.org
*Solanum lycopersicum*	725 accessions	2019	Vegetable	N.A.	N.A.

*N.A.* not available

Soybean is an economically important vegetable crop; in addition to being a source of human protein, it is an important source of vegetable oil. *Glycine soja* is the closest wild relative to cultivated soybean (*Glycine max*). The *G. soja* pan-genome was the first horticultural pan-genome released, which occurred in 2014 and consisted of seven wild accessions^85^ (Table 2). This pan-genome revealed that, when more genomes were added, the number of shared genes decreased, and in contrast, the number of total genes increased when more genomes were added. In addition, this pan-genome confirmed that a single reference genome does not adequately represent the genomic and genetic diversity of a species. Because the reference genome of *G. soja* was not previously available, those researchers assembled all seven genomes with the de novo assembly method, but this method was not adopted by subsequent researchers.

Assembly of the *B. oleracea* pan-genome^86^ is another early trial in the genomic research of horticultural plants (Table 2). It is relatively small, created using nine morphologically diverse varieties (covering two cabbage, one broccoli, one brussels sprout, one kohlrabi, two cauliflowers, and one kale plant) and a wild relative, *Brassica macrocarpa*. Through the analyses of this pan-genome, we observed that 20% of genes are absent in some cultivar(s), and there are presence–absence variations (PAVs), including those related to major agronomic traits. This is a pioneering study that provided assembled pan-genome contigs, pan-genome annotations, and the GBrowse tool, available at http://brassicagenome.net.

Pepper plants are important vegetable plants with distinct fruit morphologies. The pepper pan-genome has been generated for the pepper genus *Capsicum*^87^. This pan-genome consists of 5 species and 383 cultivars, all of which have 15 chromosomes. In addition to the comparison of PAVs among this large amount of pepper cultivars, the pan-genome is also useful in linking the association between important agronomic traits and corresponding genes. These valuable pan-genome data and JBrowse and other search tools are available (www.pepperpan.org:8012).

Sunflower plants provide seed that can be used for cooking oil and serve as popular ornamentals. The sunflower pan-genome was created by sequencing 493 accessions, including cultivars, landraces, and wild relatives^5^. A total of 61,205 genes have been identified within the gene set of the sunflower pan-genome. Via the aid of this pan-genome, the understanding of the evolutionary history of sunflower species has significantly improved, and genes linked to biotic stress resistance have been identified^5^. Although pan-genome data can be found in the sunflower genome database (www.sunflowergenome.org), no publicly accessible tool has been built to date (accessed March 31, 2019).

Reference genome sequences are necessary to identify genes and to understand evolutionary trajectory. However, a pan-genome can help to uncover additional details. For example, relying on the tomato genome sequence, researchers mapped only several genes and pathways controlling fruit ripening^28^. These flesh- and flavor-related genes are the best targets in breeding. Moreover, genome sequences allow comprehensive and systematic analyses of fruit biology. Furthermore, via the sequencing of a tomato population and analysis of its pan-genome consisting of 725 accessions, the genes selected during domestication and quality improvement were identified^88^. Thus a pan-genome not only improves our understanding of crop evolution but also is useful for the discovery of novel genes and breeding.

## Data storage and visualization

In addition to comprehensive plant-centric databases such as Phytozome (https://phytozome.jgi.doe.gov) and EnsemblPlants (http://plants.ensembl.org), 27 horticultural plant-specific genome databases have been constructed (Table 3). Among these, 22 provide data for downloading. Some databases are freely accessible to all users, while others provide only limited access to specific data or users. For example, the Genome Database for Rosaceae^89^ requires user registration and a login to access the breeding data.

**Table 3:** List of horticultural plant-centric genome databases

Database name	Covered species	Tools
Herbal Medicine Omics Database	*Calotropis gigantea*	BLAST
(herbalplant.ynau.edu.cn)	*Catharanthus roseus*	GBrowse
	*Rhodiola rosea*	
	*Gastrodia elata*	
	*Eucommia ulmoides*	
	*Camptotheca acuminata*	
	*Ginkgo biloba*	
	*Dioscorea rotundata*	
	*Panax ginseng*	
	*Punica granatum*	
	*Boea hygrometrica*	
	*Jatropha curcas*	
	*Glycyrrhiza uralensis*	
	*Cannabis sativa*	
	*Macleaya cordata*	
	*Mentha longifoli*	
	*Erigeron breviscapus*	
	*Panax notoginseng*	
	*Moringa oleifera*	
	*Lepidium meyenii*	
	*Dendrobium officinale*	
	*Salvia miltiorrhiza*	
Genome Database for Rosaceae (GDR)	*Fragaria vesca*	Breeding Information Management System
(www.rosaceae.org)	*Fragaria* x *ananassa*	BLAST+
	*Malus* x *domestica*	Breeders Toolbox
	*Prunus armeniaca*	GDRCyc
	*Prunus avium*	JBrowse
	*Prunus cerasus*	MapViewer
	*Prunus dulcis*	Pathway Inspector
	*Prunus persica*	Primer3
	*Prunus serotina*	Sequence Retrieval
	*Pyrus communis*	Synteny Viewer
	*Rubus occidentalis*	
Sol Genomics Network	*Solanum pennellii*	BLAST
(solgenomics.net)	*Solanum lycopersicoides*	VIGS Tool
	*Nicotiana attenuata*	Alignment Analyzer
	*Nicotiana benthamiana*	Tree Browser
	*Nicotiana tabacum*	Genome Browser (JBrowse)
	*Petunia axillaris*	Comparative Map Viewer
	*Petunia inflata*	CAPS Designer
	*Solanum pimpinellifolium*	solQTL: QTL Mapping
	*Solanum lycopersicum*	In Silico PCR
	Solanum tuberosum	Tomato Expression Atlas (TEA)
	*Solanum phureja*	Tomato Expression Database (TED)
	*Capsicum annuum*	SolCyc Biochemical Pathways
	*Petunia axillaris*	Coffee Interactomic Data
	*Petunia* x *hybrida*	SGN Ontology Browser
	*Petunia integrifolia* var inflata	Breeders Toolbox
		FTP Site
		Download Gene Sequences
		Clones, Arrays, Unigenes and BACs
		Unigene Converter
Citrus Genome Database (CGD)	*Citrus clementina*	Breeding Information Management System
(www.citrusgenomedb.org)	*Citrus ichangensis*	BLAST+
	*Citrus sinensis*	Breeders Toolbox
	*Citrus reticulata*	GDRCyc
	*Citrus maxima*	JBrowse
	*Citrus medica*	MapViewer
	*Poncirus trifoliata*	Pathway Inspector
	*Atalantia buxifolia*	Primer3
		Sequence Retrieval
		Synteny Viewer
Cool Season Food Legume Database (CSFL)	*Cicer arietinum*	JBrowse
(www.coolseasonfoodlegume.org)	*Cicer reticulatum*	PathwayCyc
	*Vicia faba*	Breeding Information Management System
	*Pisum sativum*	MapViewer
	*Lens culinaris*	Synteny Viewer
		BLAST+
Cucurbit Genomics Database (CuGenDB)	*Cucumis sativus*	BLAST
(cucurbitgenomics.org)	*Cucumis melo*	JBrowse
	*Citrullus lanatus*	Batch Query
	*Cucurbita maxima*	Synteny Viewer
	*Cucurbita moschata*	CMAP
	*Cucurbita pepo*	CucurbitCyc
	*Lagenaria siceraria*	Pathway enrichment
		GO enrichment
		Gene classification
Banana Genome Hub	*Musa acuminata* DH-Pahang	BLAST
(banana-genome-hub.southgreen.fr)	*Musa acuminata* Banksii	JBrowse
	*Musa acuminata* Zebrina	GBrowser
	*Musa acuminata* Calcutta 4	Generic Maps
	*Musa balbisiana* PKW	Gene Family
	*Musa Itinerans*	Chromosome viewer
	*Musa schizocarpa*	Transcriptomic Search
		Design primer
		Ontology Browser
		Dotplot
Brassica database (BRAD)	*Brassica rapa*	BLAST
(brassicadb.org)	*Brassica juncea*	Gbrowse
	*Brassica napus*	Markers and Maps
	*Brassica oleracea*	Gene families
		Glucosinolate genes
		Anthocyanin genes
		Resistance genes
		Flower genes
		Transcription factors
		Auxin genes
		Phenotypes
		People/Labs
Pepper Pangenome Browser (PepperPan)	*Capsicum annuum*	Generic genome browser
(www.pepperpan.org:8012)	*Capsicum baccatum*	
	*Capsicum frutescens*	
Cofffee Genome Hub (CGH)	*Coffea canephora*	Advanced Search
(www.coffee-genome.org/coffeacanephora)	*Coffea arabica*	Chromosome Viewer
		Gene annotation
		Gene Expression
		Gene Families
		Genetic Map
		Primer Blaster
		Primer Designer
		SNPs
		Blast
		JBrowse
		GBrowser
Viggs	Vigna marina subsp. oblonga	Gbrowse
(viggs.dna.affrc.go.jp)	*Vigna angularis*	BLAST
	Vigna angularis (Willd.)	BLAT
	*Vigna vexillata*	
Cannabis genome project (CCBR)	*Cannabis sativa*	BLAST
(genome.ccbr.utoronto.ca/cgi-bin/hgGateway)		GBrowser
		Design primer
Carnation DB	*Dianthus caryophyllus*	BLAST
(carnation.kazusa.or.jp)		
HopBase	*Humulus lupulus*	BLAST
(hopbase.cgrb.oregonstate.edu)		Gbrowse
Medicago truncatula Genome Database (MTGD)	*Medicago truncatula*	JBrowse
(www.medicagogenome.org)		BLAST
		Web Services
		CMap (LegumeInfo.org)
		GO Analysis
		InterPro Annotations
Tea Plant Information Archive (TPIA)	*Camellia sinensis*	BLAST
(tpia.teaplant.org/)		Gbrowse
		Pathway
		Correlation Analysis
		Function Erichment
		Batch Retrival
Mulberry Genome Database (MorusDB)	*Morus notabilis*	Transposable Element Analysis
(morus.swu.edu.cn/morusdb)		Horizontal Gene Transfer Analysis
		Ortholog and Paralog Group Analysis
		BLAST
		WEGO
		HMMER
		Browse GO
		Search GO
		Find Motifs
Pear Genome Project	*Pyrus* *bretschneideri*	Download
(peargenome.njau.edu.cn)		
Radish Genome database	*Raphanus sativus*	BLAST
(www.radish-genome.org/)		Gbrowse
		Expression
CsiDB	*Citrus sinensis*	Gene Search
(citrus.hzau.edu.cn)		BLAST
		GBrowser
		PPI
		Pathway
Mint Genomics Resource	*Mentha longifolia*	BLAST
(langelabtools.wsu.edu/mgr/organism/Mentha/longifolia)		Gbrowse
		Pathway
CeleryDB	*Apium graveolens*	BLAST
(apiaceae.njau.edu.cn)		GBrowser
		Transcription factors
CarrotDB	*Daucus carota*	BLAST
(apiaceae.njau.edu.cn/)		Gbrowse
		Transcription factors
		Germplasm Resources Collection
Banana Genome Hub	*Musa acuminata* DH-Pahang	BLAST
(banana-genome-hub.southgreen.fr)	*Musa acuminata* Banksii	JBrowse
	*Musa acuminata* Zebrina	GBrowser
	*Musa acuminata* Calcutta 4	Generic Maps
	*Musa balbisiana* PKW	Gene Family
	*Musa Itinerans*	Chromosome viewer
	*Musa schizocarpa*	Transcriptomic Search
		Design primer
		Ontology Browser
		Dotplot
Brassica database (BRAD)	*Brassica rapa*	BLAST
(brassicadb.org)	*Brassica juncea*	Gbrowse
	*Brassica napus*	Markers and Maps
	*Brassica oleracea*	Gene families
		Glucosinolate genes
		Anthocyanin genes
		Resistance genes
		Flower genes
		Transcription factors
		Auxin genes
		Phenotypes
		People/Labs
Pepper Pangenome Browser (PepperPan)	*Capsicum annuum*	Generic genome browser
(www.pepperpan.org:8012)	*Capsicum baccatum*	
	*Capsicum frutescens*	
Cofffee Genome Hub (CGH)	*Coffea canephora*	Advanced Search
(www.coffee-genome.org/coffeacanephora)	*Coffea arabica*	Chromosome Viewer
		Gene annotation
		Gene Expression
		Gene Families
		Genetic Map
		Primer Blaster
		Primer Designer
		SNPs
		Blast
		JBrowse
		GBrowser
Viggs	Vigna marina subsp. oblonga	Gbrowse
(viggs.dna.affrc.go.jp)	*Vigna angularis*	BLAST
	Vigna angularis (Willd.)	BLAT
	*Vigna vexillata*	
Cannabis genome project (CCBR)	*Cannabis sativa*	BLAST
(genome.ccbr.utoronto.ca/cgi-bin/hgGateway)		GBrowser
		Design primer
Carnation DB	*Dianthus caryophyllus*	BLAST
(carnation.kazusa.or.jp)		
HopBase	*Humulus lupulus*	BLAST
(hopbase.cgrb.oregonstate.edu)		Gbrowse
Medicago truncatula Genome Database (MTGD)	*Medicago truncatula*	JBrowse
(www.medicagogenome.org)		BLAST
		Web Services
		CMap (LegumeInfo.org)
		GO Analysis
		InterPro Annotations
Tea Plant Information Archive (TPIA)	*Camellia sinensis*	BLAST
(tpia.teaplant.org/)		Gbrowse
		Pathway
		Correlation Analysis
		Function Erichment
		Batch Retrival
Mulberry Genome Database (MorusDB)	*Morus notabilis*	Transposable Element Analysis
(morus.swu.edu.cn/morusdb)		Horizontal Gene Transfer Analysis
		Ortholog and Paralog Group Analysis
		BLAST
		WEGO
		HMMER
		Browse GO
		Search GO
		Find Motifs
Pear Genome Project	*Pyrus* *bretschneideri*	Download
(peargenome.njau.edu.cn)		
Radish Genome database	*Raphanus sativus*	BLAST
(www.radish-genome.org/)		Gbrowse
		Expression
CsiDB	*Citrus sinensis*	Gene Search
(citrus.hzau.edu.cn)		BLAST
		GBrowser
		PPI
		Pathway
Mint Genomics Resource	*Mentha longifolia*	BLAST
(langelabtools.wsu.edu/mgr/organism/Mentha/longifolia)		Gbrowse
		Pathway
CeleryDB	*Apium graveolens*	BLAST
(apiaceae.njau.edu.cn)		GBrowser
		Transcription factors
CarrotDB	*Daucus carota*	BLAST
(apiaceae.njau.edu.cn/)		Gbrowse
		Transcription factors
		Germplasm Resources Collection

Visualization of genomic data of horticultural plants is challenging due to the heterogeneous nature of the different types of data. GBrowse^90^ and JBrowse^91–93^ are powerful tools that provide a visualization of various levels of genomic features. The availability of genomic analysis tools also varies greatly among databases. BLAST-related tools such as NCBI-BLAST^94^ and viroBLAST^95^ are provided by some databases for homologous sequence searches and sequence comparisons. Gene query tools can help to obtain details of genes such as their sequence, annotation, and expression. HMMER^96^ searches allow the inference and extraction of gene families from genomes in the database. Syntenic tools allow the identification and visualization of genome-wide syntenic relationships across genomes. The BioCyc tools (https://biocyc.org) allow users to navigate individual pathways or the whole metabolic map of a genome for functional analyses^97^.

The Genome Database for Rosaceae (GDR), which was developed by the main bioinformatics laboratory at Washington State University^89^, is well known among the Rosaceae research community and even the plant research community. It covers the genome sequences of 18 Rosaceae species (*Fragaria vesca*, *F. ananassa*, *F. iinumae*, *F. nipponica*, *F. nubicola*, *F. orientalis*, *Malus domestica*, *Potentilla micrantha*, *Prunus avium*, *Prunus domestica*, *Prunus dulcis*, *Prunus persica*, *Prunus yedoensis*, *Pyrus bretschneideri*, *Pyrus communis*, *Rosa chinensis*, *Rosa multiflora*, and *Rubus occidentalis*), which are categorized into seven genera: *Fragaria*, *Malus*, *Potentilla*, *Prunus*, *Pyrus*, *Rosa*, and *Rubus*. To facilitate online analyses, a series of tools are provided, including genomic tools (BLAST+, JBrowse, Primer3, Sequence Retrieval, MapViewer, Synteny Viewer), metabolomic tools (GDRcyc, Pathway Inspector), and breeding tools (Breeding information Management System (BMS), Breeders Toolbox). The same team at Washington State University also developed a series of horticultural plant-themed databases, including the Citrus Genome Database, Cool-Season Food Legume Crop Database resources, and Genome Database for Vaccinium (GRIN). All these databases share a similar data process standard and have built-in bioinformatics tools.

The Sol Genomics Network (SGN)^98^, a database of Solanaceae genomic and phenotypic data and tools, was developed by Mueller’s team from the Boyce Thompson Institute for Plant Research and Cornell University. The SGN includes 11 genomes: those of *Solanum lycopersicum*, *S. lycopersicoides*, *S. pimpinellifolium*, *S. tuberosum*, *S. pennellii*, *Capsicum annuum*, *Nicotiana attenuata*, *N. benthamiana*, *N. tabacum*, *Petunia axillaris*, and *P. inflata*. These species are categorized into four economically important genera: *Solanum*, *Capsicum*, *Nicotiana*, and *Petunia*. For online analyses of genomic sequences, BLAST, Alignment Analyzer, Tree Browser, and VIGS tools are available. For mapping of various data, JBrowse, Comparative Map Viewer, CAPS Designer, and solQTL are provided. Some tools have been developed for common molecular wet laboratory experiments, including In-Silico PCR, the Tomato Expression Atlas, and the Tomato Expression Database. Systems biology tools such as SolCyc Biochemical Pathways^99^, Coffee Interactome Data, and the SGN Ontology Browser are provided. The Breeders Toolbox was developed for breeders. The same team also developed a series of horticultural plant-themed databases, including the YamBase (https://yambase.org), CassavaBase (https://cassavabase.org), and MusaBase (https://musabase.org) databases. All these databases adhere to the release of genomic data before publication (the Toronto Agreement)^100^.

The Cucurbit Genomics Database (CuGenDB)^101^ currently hosts eight high-quality genome sequences corresponding to those of cucumber (*Cucumis sativus*), water melon (*Citrullus lanatus*), winter squash (*Cucurbita maxima*), pumpkin (*Cucurbita moschata*), summer squash (*Cucurbita pepo*), muskmelon (*Cucumis melo*), bottle gourd (*Lagenaria siceraria*), and silver-seed gourd (*Cucurbita argyrosperma*). The search and batch query system allow searching for sequences and annotations. To display genomic details, the JBrowse, BLAST, Gene Ontology (GO), Synteny Viewer, CAMP, and expression viewer tools are available. To display metabolic pathways, CucurbitCyc and Pathway enrichment tools are available.

The Brassica Database (BARD)^102^, a database of important *Brassica* species, covers the vegetable species *Brassica rapa* and *B.* *oleracea*, as well as the model plant *Arabidopsis* and Brassicaceae close relatives. In addition to its genomic data, the BRAD database hosts a curated list of genes involved with anthocyanins, resistance, auxin, flowering, and glucosinolates and a full list of gene families that are of considerable importance in *Brassica* research. BLAST and JBrowse tools were built for visualization of genomic data, and syntenic tools are useful for comparative analyses.

The Herbal Medicine Omics Database^103^ includes genomic, transcriptomic, pathway, and metabolomic data for medicinal plants, although the medicinal properties of some plants are recognized only in some parts of the world. In this database, hundreds of medicinal plants are included. However, the database currently provides only the BLAST and GBrowse tools for the visualization of omics data. Other collected omic data can be downloaded but cannot be analyzed or visualized online.

There are other tool-specific databases that can be very useful for the visualization and online analyses of horticultural plant genome sequences. The Plant Genome Duplication Database (PGDD)^104^ offers online analyses of gene synteny and visualization of different results, such as dot plots (macrosynteny) and local genomic comparison plots (microsynteny). The built-in Map-View tool allows mapping of a given sequence to the genomes of 47 species from the PGDD (data accessed on March 31, 2019). The Plant Duplicate Gene Database^105^ is a collection of 141 plant species and offers online analysis and visualization of duplicated genes in select species.

## Discussions and future perspectives

### The horticultural plant genome project

It is challenging to determine the exact number of species or cultivars that exist for horticultural plants. In terms of fruit-bearing plants, at least 91 species are economically important and produce fruit that are consumed (https://simple.wikipedia.org/wiki/List_of_fruits). More than 200 vegetable plants are consumed (https://simple.wikipedia.org/wiki/List_of_vegetables). The exact number of ornamentals is also unclear, as novel cultivars are produced each year. However, it has been estimated that there are >6000 ornamental cultivars (https://www.rhs.org.uk/plants/pdfs/agm-lists/agm-ornamentals-(1).pdf), and many cultivars are created and disappear each year. Up to December 2018, genome sequences had been decoded for only 181 species, accounting for only a small proportion of the total horticultural plant species. Hence, there is a strong need to sequence additional genomes for more horticultural plants that would be valuable for comparative genomics, to better understand their evolutionary history, and to possibly make genetic modifications to better utilize these plant species.

**Fig. 2:**
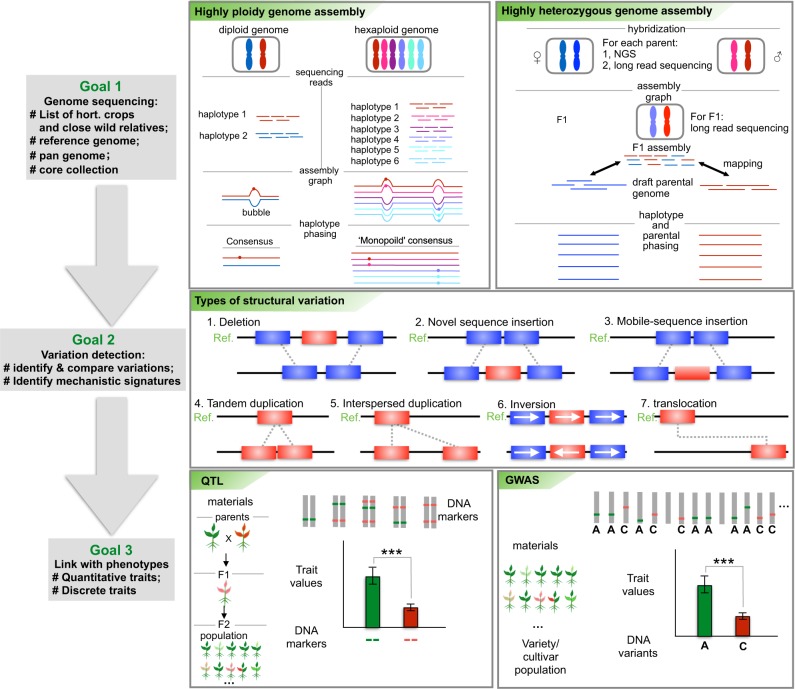
The proposed roadmap to the horticultural plant genome project (HPGP). The first goal of HPGP is to generate all reference genome sequences for horticultural plants, after which pan-genomes and core collections will be generated as a gene bank for horticultural plants. Two recently developed methods would be applied to decode the highly ploidy and highly heterozygous horticultural genomes. The second goal is to detect the various genomic variations within a pan-genome. In addition, the mechanistic signatures leading to the variations would be explored. The third goal is to link the phenotypes with the genomic regions. Two methods would be applied: the quantitative trait locus (QTL) method to correlate genomic variations with a quantitative trait and the genome-wide association study (GWAS) method to associate genomic variation with many genomic variations from different individuals ***p < 0.001

Here we propose a horticultural plant genome project (HPGP) with three goals (Fig. 2). The first goal of the HPGP is to generate reference genome sequences for all horticultural plants, after which pan-genomes and core collections would be generated as genetic banks for horticultural plants. Two recently developed genome assembly methods could be applied to decode highly ploidy^71^ and highly heterozygous^106–108^ horticultural genomes. The second goal is to identify the various genomic variations within a pan-genome. In addition, the mechanistic signatures leading to the variations would be explored. The third goal is to link the phenotypes to the genomic regions. Two methods would be applied: quantitative trait locus methods to correlate genomic variations with a quantitative trait and genome-wide association study methods to associate genomic variation with many genomic variations from different individuals^109,110^. The good news is that the Earth Genome Project and the 1000-Plant Genome Project will accelerate the genome sequencing process of horticultural plants.

The timeline for obtaining the genome sequences of all horticultural plants at both draft and reference scales (goal one of the HPGP) would be short—within 3–5 years—because the cost for sequencing is dropping rapidly. However, collecting and sequencing the population definitely requires worldwide collaborations and would take >10 years. The second goal is to analyze the genomic variations to identify the mechanistic signatures within a population, which is also time consuming and would be gradually achieved. The third goal is an advanced step that occurs after or concurrently with the second goal. Although these last two goals appear to be enormous challenges, we are confident in the ability to achieve most of these two goals in model horticultural plants such as the tomato, cucumber, and strawberry in the coming years.

In addition, the quality of assembly and annotation of existing reference genomes of horticultural plants need to be further improved. Although a few tools such as BUSCO^111^ and CEGMA^112^ have been widely used to evaluate the quality of genome annotations, a good standard is still not available for the systematic evaluation of the quality of genome assemblies. As a result, the quality of the genome assemblies is very uneven and is sometimes related to the complexity or heterozygosity of the taxa. This situation is changing as sequencing platforms are being upgraded. For example, since the first apple genome sequence was released in 2010 based on next-generation sequencing technology^15^, an improved version produced by next-generation sequencing (NGS) and PacBio technologies was released in 2016^113^. The third improved version of the apple genome, which was obtained using a combination of NGS, PacBio, and Bionano technologies, was released in 2017^114^. The fourth improved version was released in 2019, based on the utilization of NGS, PacBio, and Hi-C technologies^27^. In the future, the quality of the reference genome should reach certain minimal standards upon which the community can agree, similar to the proposal for bacteria and archaea^115^, thereby leading to more accurate pan-genome analyses and biotechnology.

Storage and access of genomic data constitute another problem concerning horticultural biologists and bioinformatics scientists. For access to genome sequences and raw sequencing data, a number of public databases are usually the first choice of researchers due to the nature of their stability, low cost, and ease of access. The well-known public databases include the NCBI (https://ncbi.nlm.nih.gov), EMBL (www.embl.org), CNGB (www.cngb.org), BIGD (bigd.big.ac.cn), DDBJ (www.ddbj.nig.ac.jp), GigaDB (gigadb.org), Dryad (www.datadryad.org), and Phytozome (https://phytozome.jgi.doe.gov) databases. To share these data with worldwide researchers, we encourage the release of data before publication, as was suggested by the Toronto Agreement in 2009^100^.

### The need for a horticultural plant-centric database

Unlike agricultural plants, horticultural plants share multiple features. For example, plant growth requires controlled conditions with specific equipment or facilities; plants generally need grafting, postharvest treatment, and a long juvenile phase; and plants usually undergo asexual reproduction and have unique specialized metabolism. All of these concerns make it hard to study these traits in model plants or via regular tools. Uniting the various omic data and the development of novel tools for horticultural plants are needed. Moreover, aside from the comprehensive plant databases and the 27 horticultural plant-specific databases mentioned above, there is still an increasing need to find and compare an increased amount of data for horticultural plants. However, horticultural biologists usually need to frequently deal with breeders; thus the need to create a comprehensive horticultural database to meet the interests of basic biologists and breeders is largely required. Such a database should cover as many horticultural plant genomes as possible and should provide an integrated set of bioinformatics tools. We believe that, in the future, the need for such a comprehensive database of all horticultural plants will satisfy additional horticulture researchers and breeders.

Given the advancement of sequencing technologies and reduced costs, the genome sequencing data of horticultural plants are accumulating rapidly. The storage, analyses, and sharing of large collections of genome sequencing data are becoming even more laborious and time consuming. The integrative analysis of various omic data, such as genomic, transcriptomic, metabolomic, phenomic, and breeding data, have become a major challenge for many horticultural biologists and requires coordinated efforts of scientists from different fields. For data processing and visualization, we recommend using BioMart tools, which could be easily built into a database. For database construction, we suggest following the template of the Tripal series (www.tripal.infor)^8^. Finally, we believe that, with a fostered collaboration of the horticultural community, the HPGP and subsequent knowledge and experiences will greatly benefit biology researchers and breeders.
